# Congenital infantile fibrosarcoma: a rare tumor dermatologists should know about^☆^

**DOI:** 10.1016/j.abd.2020.12.020

**Published:** 2022-09-06

**Authors:** Luciana Baptista Pereira, João Renato Vianna Gontijo, Marcelo de Mattos Garcia, Karine Corrêa Fonseca

**Affiliations:** a Dermatology Service, Hospital das Clínicas, Faculty of Medicine, Universidade Federal de Minas Gerais, Belo Horizonte, MG, Brazil; b Department of Dermatology, Faculty of Medicine, Universidade Federal de Minas Gerais, Belo Horizonte, MG, Brazil; a Dermatology Service, Hospital das Clínicas, Faculty of Medicine, Universidade Federal de Minas Gerais, Belo Horizonte, MG, Brazil; b Dermatology Service, Hospital Mater Dei, Belo Horizonte, MG, Brazil; a Axial Medicina Diagnóstica, Belo Horizonte, MG, Brazil; b Hospital UNIMED, Belo Horizonte, MG, Brazil; Hospital das Clínicas, Faculty of Medicine, Universidade Federal de Minas Gerais, Belo Horizonte, MG, Brazil

Dear Editor,

A seven-month-old female patient presented with a history of a congenital, violaceous, fast-growing lesion located on the right plantar surface. Dermatological examination disclosed the presence of a firm spherical tumor, with dilated vessels on the surface, and central ulceration with friable, bleeding tissue, and hematic crusts (Fig. 1A). The child developed severe anemia (hemoglobin of 4.4 g/dL), requiring a blood transfusion. The platelet count was normal. Histopathology was suggestive of kaposiform hemangioendothelioma. Treatment with oral prednisolone (2 mg/kg/day) was started but was interrupted after one month, due to lack of a response (Fig. 1B).

**Figure 1:**
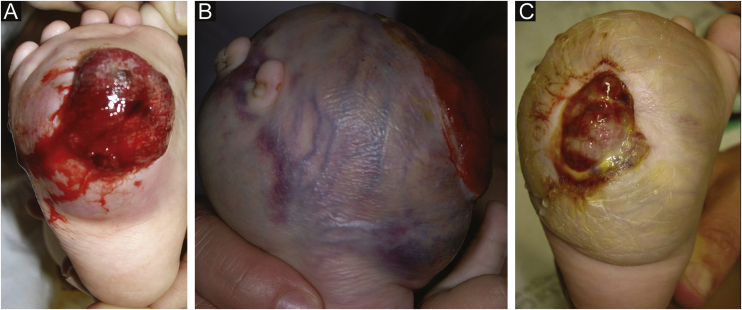
(A) Ulcerated and bleeding tumor mass. (B) After 13 weeks, significant increase in size (before the chemotherapy). (C) Tumor and ulcer reduction after adjuvant chemotherapy and before amputation.

Magnetic resonance imaging (MRI) and magnetic resonance angiography (MRA) disclosed a well-vascularized solid mass, with the involvement of the underlying muscles and extending to the anterior aspect of the foot. Diffuse contrast enhancement was observed throughout the lesion, with no signs of arteriovenous shunts or a cluster of tortuous vessels (nidus), thus ruling out the diagnosis of a vascular tumor, including kaposiform hemangioendothelioma (Figs. 2A and 2B). A second biopsy was performed, revealing a hypercellular fusiform tumor. Immunohistochemistry was positive for vimentin and negative for CD31, CD34, factor VIII, desmin, MyoD1, myogenin, CD99 and EMA, indicating the diagnosis of congenital infantile fibrosarcoma (CIF).

**Figure 2:**
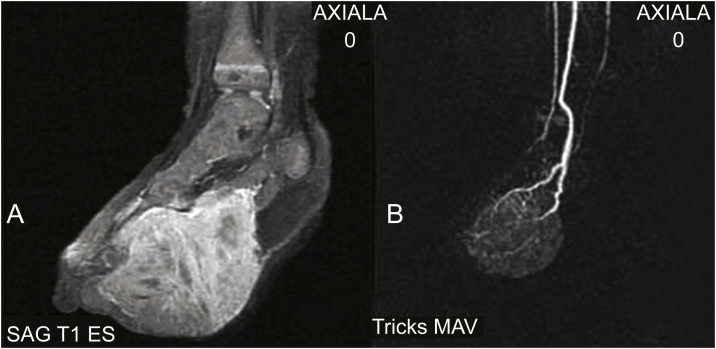
(A) MRI identifying an expansive mass with diffuse contrast enhancement. (B) MRA showing an expansive lesion supported by vascular structures. There is no evidence of arteriovenous fistulas or nidus.

The patient was submitted to neoadjuvant chemotherapy (vincristine, actinomycin-D and cyclophosphamide) to reduce tumor size (Fig. 1C), followed by amputation of the foot. There are no signs of recurrence or metastasis at five years of follow-up.

CIF is a rare malignant tumor of childhood; however, it is the most common soft tissue sarcoma in children under one year of age.^1^ This highly vascularized congenital tumor is difficult to clinically differentiate from vascular tumors or malformations. It may be present at birth or develop during the first five years, with approximately 80% of cases diagnosed during the first year of life.^2^

Fibrosarcomas are malignant neoplasias composed of mesenchymal fibroblasts. The infantile variant shares histopathological characteristics with adult fibrosarcoma but has a better prognosis. Although local recurrences are common, the rate of CIF metastasis is less than 10% and the ten-year survival rate is up to 90%.^3^ The extremities are more commonly affected and lesions located on the trunk, head and neck are less frequent, although they are more aggressive.^1^, ^4^ Due to the risk of local recurrence, extensive surgical resection is recommended. Surgery alone shows recurrence rates of 17% to 40%. Neoadjuvant chemotherapy reduces the risk of local recurrence and metastases.^2^, ^3^, ^5^

The histopathological findings of CIF include the proliferation of dense fusiform cells and vascularized areas. Immunohistochemistry is positive for vimentin and, in some cases, for desmin, smooth muscle actin, and cytokeratin.^4^ CIF is characterized in up to 85% of cases by a specific t(12;15) (p13:q25) chromosomal translocation encoding an ETV6-NTRK3 gene fusion.^1^, ^3^, ^4^, ^5^

The diagnosis of CIF should always be considered in the presence of a congenital, spherical, bleeding extremity tumor in children, aiming to avoid treatment delays.

## Financial support

None declared.

## Authors’ contributions

Luciana Baptista Pereira: Design and planning of the study; drafting and editing of the manuscript; collection, analysis, and interpretation of data; critical review of the manuscript; approval of the final version of the manuscript.

João Renato Vianna Gontijo: Critical review of the manuscript; drafting and editing of the manuscript; approval of the final version of the manuscript.

Marcelo de Mattos Garcia: Critical review of the manuscript; drafting and editing of the manuscript; approval of the final version of the manuscript.

Karine Corrêa Fonseca: Critical review of the manuscript; drafting and editing of the manuscript; approval of the final version of the manuscript.

## Conflicts of interest

None declared.
